# O-Phthalaldehyde Derivatization for the Paper-Based Fluorometric Determination of Glutathione in Nutritional Supplements

**DOI:** 10.3390/molecules29112550

**Published:** 2024-05-29

**Authors:** Maria Tarara, Paraskevas D. Tzanavaras, George Z. Tsogas

**Affiliations:** Laboratory of Analytical Chemistry, School of Chemistry, Faculty of Sciences, Aristotle University of Thessaloniki, GR-54124 Thessaloniki, Greece; mariatarara@chem.auth.gr (M.T.); ptzanava@chem.auth.gr (P.D.T.)

**Keywords:** paper-based analytical devices, fluorimetric determination, glutathione, UV irradiation, nutritional supplement formulations

## Abstract

Herein, a new, direct paper-based fluorimetric method is described for the quantitative determination of glutathione (GSH) molecules in nutritional supplements. Briefly, the proposed analytical method is based on the fluorescence emission resulting from the direct and selective chemical reaction of GSH molecules with the derivatization reagent that is o-phthalaldehyde (OPA) in acidic conditions at room temperature. The intensity of the emitted fluorescence on the surface of the analytical paper devices after irradiation with a lamp at 365 nm is proportional to the concentration of GSH and is measured using a smartphone as the detector. This methodology, which is suitable for measurements in laboratories with limited resources, does not require specialized instrumentation or trained personnel. The protocol governing the proposed method is simple and easily applicable. Essentially, the chemical analyst should adjust the value of pH on the surface of the paper by adding a minimal amount of buffer solution; then, after adding a few microliters of the derivatization reagent, wait for the surface of the paper to dry and, finally, add the analyte. Subsequently, the irradiation of the sensor and the measurement of the emitted fluorescence can be recorded with a mobile phone. In the present study, several parameters affecting the chemical reaction and the emitted fluorescence were optimized, the effect of interfering compounds that may be present in dietary supplements was examined, and the stability of these paper sensors under different storage conditions was evaluated. Additionally, the chemical stability of these paper devices in various maintenance conditions was studied, with satisfactory results. The detection limit calculated as 3.3 S/N was 20.5 μmol L^−1^, while the precision of the method was satisfactory, ranging from 3.1% (intra-day) to 7.3% (inter-day). Finally, the method was successfully applied to three different samples of dietary supplements.

## 1. Introduction

Glutathione (GSH) is a simple sulfur compound, a thiol, and is noted as an essential antioxidant in many food samples such as plants, formed of cysteine, glutamic acid, and glycine [1]. GSH, as an essential antioxidant and enzymatic cofactor, plays a significant role in many important biological reactions and many physiological and biochemical processes of the human body, including signal transduction, gene expression, protein glutathionylation, and nitric oxide (NO) metabolism [2]. Insufficient GSH concentrations in the human body have been associated with many pathological conditions including diabetes and Parkinson’s disease [3]. As an outcome, it is possible that preserving the body’s endogenous GSH levels is important in maintaining health and moderating disease symptoms. However, clear interactions between low GSH levels and disease risk remain to be clarified [4].

From the analytical chemistry point of view, a search in the scientific bibliography has revealed a persistent interest in developing new methods for the analytical determination of GSH with many instrumentation techniques including high-performance liquid chromatography [5,6,7], zone fluidics [8], capillary electrophoresis [9], electroanalysis [10], resonance light scattering [11], mass spectrometry [12,13], and chemiluminescence [14,15]. The most popular methods for GSH determination are colorimetric procedures [16,17,18,19,20], fluorescence methods [21,22,23,24] with the addition of nanostructures, and spectrophotometric or fluorimetric methods after derivatization [25,26,27]. 

Recently, various enzyme-like mimic systems have been developed for the quantitative determination of GSH. Based on such systems, many simple colorimetric methods have been accomplished for the analysis of GSH including iron oxychloride (FeOCl) nanosheets [28], human serum albumin (HSA)-templated MnO_2_ nanosheets [29], Fe_3_O_4_/CNDs hybrid nanoparticles (NPs) [30], CoFe_2_O_4_ modified with MoS_2_, to form a MoS_2_@CoFe_2_O_4_ polymer [31], perylene diimide (PDI) functionalized CeO_2_ nanoparticles [32], Erythrocyte-like copper sulfide nanoparticles (Cu1.8S NPs) [33], and a magnetic supported gold-copper bimetallic organic framework (Fe_3_O_4_@Au/Cu-MOF) [34]. Although these methods are sensitive and reproducible, the synthetic route of the proposed nanomaterials is often composite and time consuming. 

With this motivation, our research group tried to develop a new, simple, and fast analytical method for the quantitative determination of GSH. Different derivatization reagents have been studied for the fluorimetric determination of GSH, including o-phenylenediamine (OPD) [35], 1,8-naphthalimide (NI) [36], and ortophthalaldehyde (OPA). OPA is an established derivatization reagent for the fluorimetric determination of GSH [8,37]. Reduced GSH reacts with OPA to form a stable, highly fluorescent tricyclic derivate at pH 8, while other essential amino acids like L-histidine (His) react at a higher alkaline environment [38]. Additionally, paper-based analytical devices (PADs) have been developed in the past few years, at extremely fast rates, as alternative platforms for the determination of thiols based on their significant advantages, including low cost, portability, and minimal instrumentation demands [38,39,40]. Moreover, electronic imaging devices including flatbed scanners [41] and smartphones [42,43] have been used in the last few years for the analytical determination of GSH.

Herein, a new paper-based fluorimetric method is described for the first time for the quantitative determination of GSH. The method does not require any nucleophilic reagent for the derivatization of GSH with OPA and the experiments are conducted in an alkaline environment at room temperature. After the chemical reaction between GSH and OPA, the paper platforms are irradiated at 365 nm for a limited time with a UV lamp and the intense fluorescence emitted is immediately determined by taking a photo using a modern smartphone, operating as a simple fluorimeter. This method includes an easily applicable, equipment-free experimental procedure with a smartphone as a probe, making it suitable for GSH determination in diary supplements analysis. The method was used for the determination of GSH in food supplement formulations with very good results. 

## 2. Results and Discussion

### 2.1. Optimization Procedures

The influence of the experimental parameters and the applicability of the paper-based procedure presented herein, for the selective fluorimetric determination of GSH in nutritional supplements, was investigated in detail. Each parameter was studied through individual experiments, keeping invariable all other parameters affected by this methodology. After setting the optimum value for the first parameter, we studied the next one, and this was the motif followed for all optimization parameters.

#### 2.1.1. Effect of OPA Concentration

**Figure 1:**
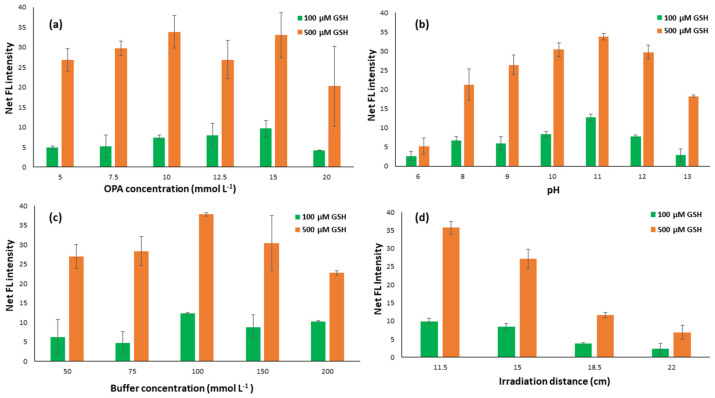
Optimization of (**a**) OPA concentration (pH 12, buffer concentration 100 mM, irradiation distance 11.5 cm), (**b**) pH (OPA 15 mM, buffer concentration 100 mM, irradiation distance 11.5 cm), (**c**) buffer concentration (OPA 15 mM, pH 11, irradiation distance 11.5 cm), and (**d**) irradiation distance (OPA 15 mM, pH 11, buffer concentration 100 mM). Error bars are the standard deviation for *n* = 3.

The concentration of the derivatization reagent is significant as it designates the excess or not of OPA molecules and consequently the intensity of the emitted fluorescence and the accuracy of the method. Consequently, it was the first parameter studied to optimize the method in a concentration range between 5 and 20 mmol mL^−1^, with the optimal fluorescence signal (sample fluorescence minus blank fluorescence) being observed for a concentration of 15 mmol mL^−1^, as is depicted in Figure 1a. The fluorescence of the blank sample emitted on the surface of the paper substrate at any OPA concentration is practically negligible and this is because the absence of any nucleophilic reagent gives selectivity characteristics to the specific reaction [44]. In OPA concentrations higher than this, a decrease in absolute fluorescence was observed, possibly due to the presence of a second OPA molecule that reacts with the analyte to form a non-fluorescent product, as has been observed in previous studies, while on the contrary, at very low concentrations, no OPA molecules are sufficient for the formation of the fluorescent product [45].

#### 2.1.2. Effect of pH and Buffer Concentration

The study of the pH effect in the proposed method was conducted from weakly acidic to strongly alkaline values (6.0 to 13.0) due to the existence of the amino groups in the amino acid molecules. All amino acids without exception are distinguished by pKa values that vary between 8.7 and 10.7, while, in particular, GSH presents a pKa value equal to 9.65, so the derivatization reactions with OPA molecules were carried out in alkaline conditions. As can be easily seen from the results (Figure 1b), the derivatization of the analyte with the OPA on the paper substrate is favored for a pH value equal to 11, something that has also been clarified in previous studies, in which it was determined as the optimal pH area between 10 and 12 [46,47].

However, apart from the value of the pH of the buffer solution, its concentration has also played an important role, according to previous studies, in the successful derivatization of various amino acids with OPA [48,49]. Therefore, it was decided to determine the optimal value of the concentration of the buffer solution and the study was carried out for concentrations ranging between 50 and 200 µM. The optimal concentration of the buffer solution, as can be seen in Figure 1c, was found to be equal to 100 µM, so this was the concentration chosen for the continuation of the experiments.

#### 2.1.3. Effect of UV Irradiation Distance

The irradiation distance played a crucial role because of the amount of radiation received on the paper surface in the fluorimetric determination of GSH in the proposed method. An irradiation lamp at 365 nm was used for this study, emitting UV radiation in close agreement with previous works confirming that the excitation wavelength of GSH is 340 to 365 nm [47,50]. Various distances from the UV lamp were studied, from 3.0 to 22.0 cm, and the distance of 11.5 cm was found to be the optimum one (Figure 1d). Distances lower than 11.5 cm were not convenient enough because the cellphone camera was exceedingly difficult to settle between the paper device and the UV beam and, thus, the 11.5 cm distance was selected during the experimental procedure.

#### 2.1.4. Effect of Reaction Time

**Figure 2:**
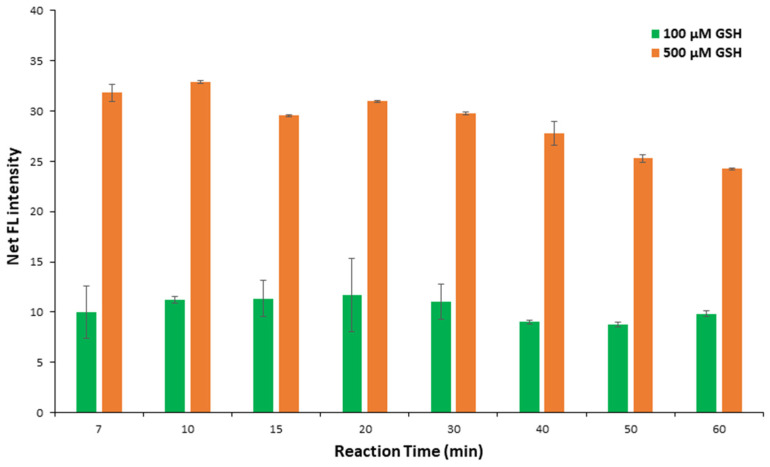
Optimization of reaction time (OPA concentration 15 mM, buffer pH 11, buffer concentration 100 mM, irradiation distance 11.5 cm). Error bars are the standard deviation for *n* = 3.

The reaction time influence was studied between 7 and 60 min. Because the analytical protocol included drying the paper devices, the shortest time to assess the results was 7 min. The fluorescence produced from the derivatization reaction between OPA and GSH is a rapid phenomenon at room temperature, concluded in a few seconds, and is eliminated over time, which comes to an agreement with previous reports [51]. Thus, the net FL signal was decreased for reaction times higher than 10 min (Figure 2). 

Therefore, the photograph was taken just after the reagents or the samples used were dry on the paper surface. The optimum reaction time was chosen to be 10 min, in an attempt to assemble the lower time for the papers to dry and the maximum fluorescence performance to be obtained.

#### 2.1.5. Effect of Reagent Sequence

The main advantage of analytical paper devices is their portability in the field, and from our previous report [38], we have decided that the order of addition of the reagents is considered a key parameter to determine the manner and order of their deposition on the surface of the paper to manufacture suitable sensors for analytical determinations. It is a given that in all field analyses, the amount of analyte is added at the end of the experimental process, as all the reactants should be present and then the determination of the analyte be made. Motivated by this certainty, it was decided to study the order of the reagent addition in two different ways: (a) buffer, OPA, and GSH; and (b) OPA, buffer, and GSH. The net FL signals decreased by 11% and 2% for GSH concentrations of 100 and 500 μM, respectively, from the first to the second reagent sequence route, and as a consequence, the first reagent addition route (buffer, OPA, and GSH) was followed. 

### 2.2. Analytical Parameters

The proposed paper-based fluorimetric method has been punctiliously validated regarding parameters such as accuracy, linearity, limits of detection (LOD), and quantification (LOQ), precision, selectivity, and stability of the PADs.

#### 2.2.1. Figures of Merit

**Figure 3:**
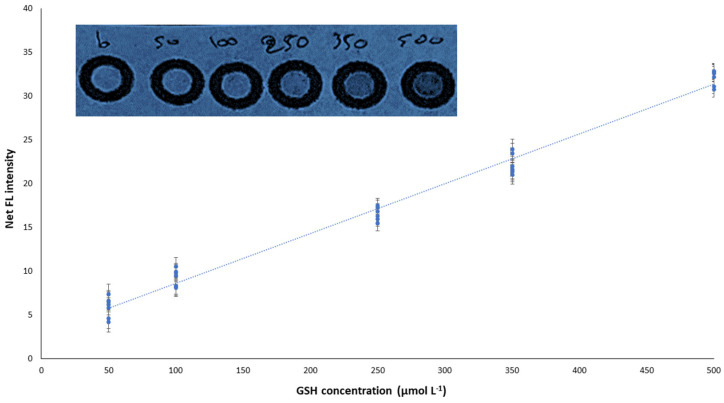
The “cumulative” calibration curve of the proposed method. Error bars are standard deviation for *n* = 3.

In terms of method validation, the proposed method rendered satisfactory linearity for GSH concentrations in the range of 50–500 μmol L^−1^ (Figure 3). Under the optimum conditions, a “cumulative” regression equation was obtained by assimilating the results from 40 standard solutions analyzed in different working days (*n* = 8), setting the calibration curve more representative by embracing any potential variations.

So, the next regression Equation was obtained:

FL = 0.057 (±0.002) [GSH] + 2.90 (±0.54), r^2^ = 0.986

where FL is the fluorescence intensity measured. 

Additionally, the within-day precision was calculated at two GSH concentration values, 100 and 350 μmol L^−1^, by repetitious measurements of different paper devices (*n* = 5). The intra-day relative standard deviation (RSD) was 6.2% and 3.1%, respectively, for these two concentration values, while the inter-day RSD was 7.3% and 4.4%, respectively. For both intra-day and inter-day precision experiments, de-ionized water was utilized as the solvent for standard solutions. 

Additionally, the LOD and LOQ were calculated as 3.3 × SDb/s and 10 × SDb/s, where SDb is the standard deviation of the intercept and s is the slope of the respective regression lines. The calculated LOD/LOQ for the determination of GSH were 20.5 and 62.2 μmol L^−1^, respectively.

#### 2.2.2. Selectivity

**Figure 4:**
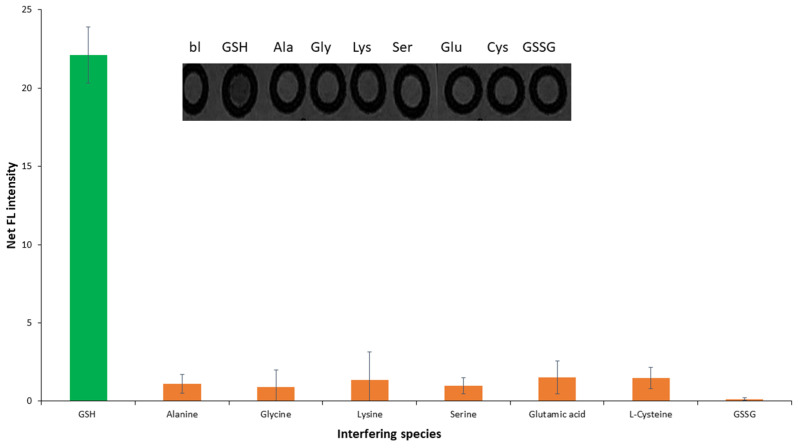
Selectivity study of the OPA-GSH reaction under the optimum experimental conditions (GSH 350 μM, OPA 15 mM, irradiation distance 11.5 cm, reaction time 10 min, buffer pH 11, and buffer concentration 100 mM). Error bars are the standard deviation for *n* = 3.

OPA could react with other molecules like amino acids and L-cysteine, but for this reaction, a nucleophilic reagent is necessary through a traditional mechanism [52]. Thus, the proposed method (without any nucleophilic compound) is selective against analytes that have primary amino groups in their molecules, providing no decrease in the sensitivity and the selectivity of the method. The selectivity of the developed paper devices was studied against the most common compounds that tend to react with OPA under the classic mechanism (glycine, alanine, serine, and lysine) and additionally against compounds that are expected to be present in GSH nutritional supplements (glutamic acid, L-cysteine, and the oxidative form of glutathione, GSSG); histidine, which reacts with OPA through the same mechanism as GSH, was examined and no significant interference was observed due to the different pH reaction value (pH = 10) [38]. GSH concentration studied for the selectivity study was kept steady at 350 μmol L^−1^, while all potential interferents were analyzed at a 10-fold higher concentration, except for L-cysteine and GSSG which were analyzed at a 5-fold higher concentration. The experimental results are shown in Figure 4 and verified the adequate selectivity of the procedure, considering the expected high levels and ratios of GSH in nutritional supplement samples.

#### 2.2.3. Stability of the Devices

A buffer solution and an OPA reagent were placed on the surface of the detection point of the paper devices and they were stored in airtight bags, protected from light at three different temperatures (room temperature, 4 °C, and −18 °C), to evaluate the portability and the stability of these platforms. The stability was tested at 350 μmol L^−1^ GSH for 2 days, 4 days, and 6 days of storage time, respectively. The stability performance of the sensors was studied at five different paper platforms for each condition per day. The experimental results as % recoveries of GSH are analyzed in Table 1. It is clear from the calculated recoveries that the paper devices are stable and, thus, ready for use even after 6 days if kept at 4 °C and protected from light.

### 2.3. Application in Nutritional Supplement Samples

Three nutritional supplement samples with different GSH content were treated as described in the Experimental Section and were analyzed in triplicates by the developed paper-based fluorimetric method, and the experimental results are gathered in Table 2, where the accuracy of the method was established based on the stated value of the GSH concentration on the packaging of the nutritional preparations studied. Further evaluation, including analysis of the same samples by an HPLC-PCD method, was conducted. The corroborative GSH concentration values and the calculated Relative Errors (%) are also included in Table 3.

## 3. Materials and Methods

### 3.1. Reagents and Solutions

L-Glutathione (GSH, 98+%) was obtained from Thermo Fisher Scientific (Kendal, Germany). Hydrochloric acid and ammonium chloride were purchased by Sigma (St. Louis, MO, USA). Analytical grade o-phthalaldehyde (OPA) was obtained from Fluka (Munich, Germany) while KH_2_PO_4_ and NaOH were from Merck (Darmstadt, Germany). Doubly de-ionized water was produced by a Milli-Q system (Millipore, Bedford, MA, USA). The standard stock solution of GSH was prepared daily at the 5000 μmol L^−1^ level in doubly de-ionized water and working solutions were prepared by the appropriate dilutions. The derivatization reagent (OPA) was prepared at an amount concentration of 15 mmol L^−1^ by firstly dissolving in 0.5 mL methanol and subsequently adding 9.5 mL water [8]. This solution was practically stable for a working period of 3–4 days in the refrigerator (4 °C) in an amber glass vial. Phosphate buffer was also prepared daily and regulated to the desired pH value by drop-wise addition of NaOH (2.0 mol L^−1^). Analytical grade glycine, glutamic acid, alanine, lysine, and serine used in the selectivity studies were supplied by Sigma (St. Louis, MO, USA), while L-cysteine was purchased by TCI (Tokyo, Japan) and glutathione disulfide (GSSG) by Fluka (Munich, Germany).

### 3.2. Apparatus

**Figure 5:**
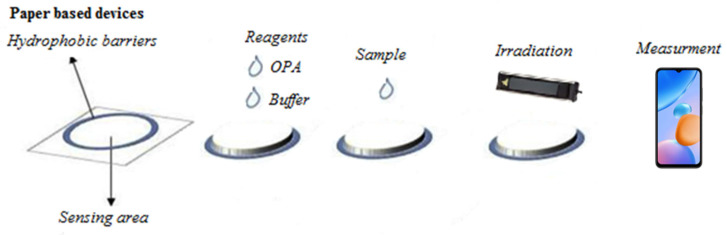
The experimental course of the method.

The paper devices made for this assay were circular in shape and created using a solid ink printer (ColorQube 8580DN Xerox, Xerox Hellas A.E.E. 350 Syngrou Avenue 17674 Kallithea, Athens, Greece) (Figure 5). The paper used was Whatman No 1 chromatography paper, while the design of the tracing funnel patterns was done with the PowerPoint program in pre-planned designs. The preparation of the buffer solutions and the adjustment of their values were done with a pH meter (Orion, Thermo Scientific Orion: Waltham, MA, USA), while the irradiation of the paper devices was carried out with the use of a UV radiation lamp that operated with a power equal to 15 W (Vilber Lourmat 115-BL, Vilber, smart imaging, BP 31—ZAC de Lamirault, Collégien, F-77601 Marne-la-Vallée cedex 3, France). The images during the whole analysis were captured with a smartphone (Xiaomi Redmi Note 10, Xiaomi store Thessaloniki, Paulou Mela 14, 54622, Thessaloniki, Greece), equipped with a 48 MP + 8 MP + 2 MP + 2 MP quad camera, containing a 48 MP wide-angle camera, an 8 MP ultra-wide-angle camera, a 2 MP macro camera, and a 2 MP depth sensor) and the fluorescence emitted was determined with the Image J program (v1.53k).

### 3.3. Fabrication of the Paper Devices

The selection of paper for the manufacture of these devices was based on the best performance reported in previous publications [53]. For the paper array to produce reproducible results, it must be uniform over its entire area, and able to rapidly absorb solutions and unknown samples, so it must have a greater mass of paper per cm. These conditions are more adequately satisfied by the chromatographic paper Whatman No. 1 with relatively high thickness and mass per area (0.18 mm, 87 gm^−2^) [53], and, thus, paper-based devices crafted from this substrate were used during the conduct of all the experiments. After designing and manufacturing these analytical devices using the solid wax printer, the wax only exists on one side of the paper. To create the hydrophobic barriers, so that the solutions do not diffuse outside the detection zone, the wax must penetrate the paper and form a barrier on its backside as well. To achieve this, these analytical arrangements should be heated in an oven for 2 min at 120 ± 5 °C. The heating time and temperature are important because this will create uniform hydrophilic zones and corresponding uniform hydrophobic barriers. In this way, devices were prepared that had an inner diameter of 4 mm (hydrophilic sensing zone), a barrier thickness of 2 mm, and an outer diameter equal to 8 mm.

### 3.4. Experimental Procedure

The experimental procedure proposed in this study is fast and easy to perform with very little consumption of chemical reagents and minimal required laboratory equipment. Briefly, the procedure followed was as follows: Adding the buffer solution (1 μL, 100 mmol L^−1^) so that the surface of the paper acquires a pH value of 11, waiting for the paper to dry (about 10 min at room temperature), and then adding the derivatization reagent (OPA, 1 μL, 15 mmol L^−1^). Finally, the GSH standard solutions or the unknown samples (1 μL) were added. After adding the analyte and drying the devices, they were placed directly (no longer than 30 s) under the 365 nm irradiation lamp, and the photograph was captured with the smartphone.

The photographs were saved as files in JPEG format (at least 300 dpi), and the Image J program was used to determine the mean intensity of the fluorescence in the red mode of the RGB color model (Figure 5). The photographs taken were opened with the ImageJ program, and the red mode of the RGB color model was selected. Then, the elliptical shape was chosen, and it was placed onto the circular detection area in such a way as to cover the total hydrophilic sensing area. This elliptical area was kept constant for all the measurements. Finally, by pressing the M (measure) button, the measurement of the fluorescence on the paper sensing zone was achieved.

The statistics used in this study were as simple as it can get. We used the Excel program (Microsoft Excel Office 2016) and by the function input, and we studied the average of our measurements and the standard deviation of each one. Then, the %RSD was calculated as the SD/mean value × 100%. 

### 3.5. Real Samples

Nutritional supplements samples containing different amounts of GSH were purchased from local drugstores. Three different food supplements that are sugar, salt, and starch-free with GSH concentrations of 25, 50, and 100 mg per vegetable capsule were tested. Due to the selectivity of the proposed method, sample preparation was non-laborious and included the following brief steps: Each pill was weighted at an analytical balance, and it was put in a volumetric flask of 50 mL. All the mass of the pill weighted (approximately 0.38–0.39 g) was diluted in de-ionized water. After the ultrasonic dissolution of the pill for 20 min, the solution was filtrated through common filter paper. Finally, a 5 to 15-fold dilution with milli-Q water, depending on the levels of GSH in the real samples, was conducted, followed by the analysis with the proposed paper-based fluorimetric method. 

## 4. Conclusions

A simple and accurate paper-based analytical method for the selective determination of GSH in nutritional supplements has been developed and validated. The PADs were effortlessly fabricated at a small cost. The excitation process was carried out by a commercially available UV lamp and the fluorimetric emission was measured by a smartphone. The fluorimetric sensor developed was sensitive enough for the quantification of the analyte in real samples with minimal treatment and successfully applied to nutritional supplements with insignificant relative errors. Additionally, the paper devices were stable at low temperatures for at least six days and were used without any drawbacks for the analysis of GSH. Finally, the developed analytical method is reliable and repeatable and permits the analysis of GSH in nutritional supplements at low micromolar levels.

## Figures and Tables

**Table 1:** Stability of the devices after the addition of buffer + OPA solutions under various storage conditions.

	Time (Days)		
	2	4	6
Temperature (°C)		Recovery %	
25	76.5 ± 6.7	73.8 ± 5.9	71.2 ± 5.5
4	94.7 ± 7.8	88.8 ± 7.3	85.2 ± 8.4
−18	73.3 ± 6.5	69.6 ± 6.0	63.9 ± 10.6

**Table 2:** Determination of GSH concentration in real samples.

Samples	Paper-Based Method GSH (μmol L^−1^) (S.D.)	GSH Concentration of the Nutritional Supplements (μmol L^−1^)	Relative Error (RE%)
Sample 1	3143 (±161)	3253	−3.4
Sample 2	4605 (±235)	4880	−5.6
Sample 3	1620 (±66)	1627	−0.4

**Table 3:** Verification of GSH concentration in real samples with an HPLC-PCD method.

Samples	Paper-Based Method GSH (μmol L^−1^) (S.D.)	GSH Concentration Determined with HPLC-PCD Method (μmol L^−1^)	Relative Error (RE%)
Sample 1	3143 (±161)	3218 (±31)	−2.4
Sample 2	4605 (±235)	4780 (±86)	−3.8
Sample 3	1620 (±66)	1649 (±18)	−1.8

## Data Availability

Data are contained within the article.
